# Attachment and Chronic Pain in Children and Adolescents

**DOI:** 10.3390/children3040021

**Published:** 2016-10-25

**Authors:** Theresa J. Donnelly, Tiina Jaaniste

**Affiliations:** 1 Department of Pain & Palliative Care, Sydney Children’s Hospital, Randwick, NSW 2031, Australia; Theresa.Donnelly@health.nsw.gov.au; 2 School of Women’s and Children’s Health, University of New South Wales, Kensington, NSW 2052, Australia

**Keywords:** attachment, chronic pain, child, adolescent

## Abstract

Although attachment theory is not new, its theoretical implications for the pediatric chronic pain context have not been thoroughly considered, and the empirical implications and potential clinical applications are worth exploring. The attachment framework broadly focuses on interactions between a child’s developing self-regulatory systems and their caregiver’s responses. These interactions are believed to create a template for how individuals will relate to others in the future, and may help account for normative and pathological patterns of emotions and behavior throughout life. This review outlines relevant aspects of the attachment framework to the pediatric chronic pain context. The theoretical and empirical literature is reviewed regarding the potential role of attachment-based constructs such as vulnerability and maintaining factors of pediatric chronic pain. The nature and targets of attachment-based pediatric interventions are considered, with particular focus on relevance for the pediatric chronic pain context. The potential role of attachment style in the transition from acute to chronic pain is considered, with further research directions outlined.

## 1. Introduction

The term attachment theory has come to loosely refer to a cluster of theories, stemming from the seminal work of Bowlby [1] and Ainsworth [2,3]. Attachment theories broadly focus on interactions between a child’s developing self-regulatory systems and their caregiver’s responses, and are believed to shed light on an individual’s cognitions, emotions and behavior throughout life, helping account for both normative and pathological patterns. The attachment system is considered to be instinctive and biologically based [1], with infants striving to maintain proximity to a caregiver in order to ensure survival needs are attended to [1,4]. The attachment system is understood to be predicated on the goal of increasing the individual’s chance of survival and is particularly activated in circumstances where there is a threat such as pain.

The goals of attachment behavior are thought to evolve as a child develops, transitioning from proximity and closeness in infancy and early childhood, through to availability and a secure base from which to explore in middle childhood and adolescence. Infants learn that cries resulting from physical discomfort and needs such as hunger are responded to by a loving caregiver. This is thought to become a template for future relationships. Hence, Bowlby [1,5,6] considered attachment as a relatively stable aspect of personality that is shaped early in life in response to the caregiver’s responsivity, consistency, and sensitivity to the child. This is subsequently influenced or reinforced via interactions with caregivers throughout childhood and adolescence [7] and significant others throughout life [8].

Research has generally supported the key premise of attachment theory, namely that early security is beneficial for the developmental adaptation and competence of children, whereas insecurity carries risks for psychopathology (for reviews see [9,10]; for meta-analysis see [11]). Moreover, there is increasing evidence that insecure attachment styles may also carry risks of greater physical symptoms, such as pain [12,13] and greater disability [14,15]. However, the vast majority of this research has been cross-sectional rather than longitudinal, thus making causal inferences difficult.

It has been proposed that an individual’s characteristic attachment behaviors are likely to be activated as a result of an illness or threat [16]. Illness, and arguably pain, may trigger an increased need for security and the wish for a close, caring other. This may be an adaptive response within the context of an injury or acute pain. However, in the context of chronic pain this can result in a range of complex and difficult behavioral interactions. Integral to attachment theory are the complex interactions between a child’s developing self-regulatory systems and the caregiver’s responses, generating a template for the child’s emotional and behavioral response patterns throughout life. These systems and interactions are also highly relevant to the chronic pain context and the resulting emotional and behavioral dynamics that become such a central part of the chronic pain experience.

The central aim of this paper is to review the available literature on attachment, specifically with regard to the potential relevance of the attachment framework on enhancing our understanding of children’s chronic pain experience and management. The paper will overview some key attachment taxonomies and factor-based attachment structures before exploring the relationship between attachment style and chronic pain. Utilizing a theoretical and empirical perspective, attachment constructs will be considered as possible vulnerability factors or maintaining factors for children’s chronic pain. This paper will also provide an overview of the literature on interventions for children and adolescents derived from attachment theory, with particular focus on the potential relevance for the pediatric chronic pain context. Possible directions for future research and clinical application will be considered.

## 2. Attachment Taxonomies

Since Bowlby [5] highlighted the importance of attachment, attachment systems have been described in terms of resultant behavioral styles reflecting the structure of two internal working models: the model of the self and the model of others. Ainsworth [3], in coding infant behavior in the context of a stressful separation from their caregiver, determined three classifications based on patterns of predictable reactions: 1) secure (exhibited protest on the departure of their caregiver, sought out the caregiver on their return); 2) anxious–ambivalent (exhibited significant distress at the departure of the caregiver and difficulty settling upon their return); and 3) avoidant (did not demonstrate distress on the departure of the caregiver and ignored the caregiver upon their return). Within this conception, a secure style reflects a positive model of both the self and of others. Both the anxious–ambivalent and avoidant styles reflect the manifestation of at least one negative working model, either of others (avoidant), or of the self in conjunction with a positive model of others (anxious-ambivalent). Main [17] instigated the addition of a fourth ‘disorganized/disoriented’ classification to reflect the use of a combination of both approach and avoidant strategies, typically associated with maltreatment.

Bartholomew and Horowitz [18] were the first to consider all possible combinations of the internal models of self and others to create four discrete factors in an adult sample utilizing self-report measures: (1) secure (self: positive, other: positive; comfortable with intimacy and autonomy); (2) pre-occupied (self: negative, other: positive; preoccupied with relationships); (3) dismissing (self: positive, other: negative; dismissing of intimacy, counter-dependent); (4) fearful (self: negative, other: negative; fearful of intimacy, socially avoidant). The significant addition of this taxonomy is the division of the earlier avoidant (negative model of the other) classification, in terms of the differentiating model of the self. Application of the four-factor structure has also been supported in adolescent populations [19,20].

While a significant portion of the literature has focused on the theoretical models espoused above, recent attention, particularly from within health care settings, has been directed toward the Dynamic Maturational Model of Attachment [21,22,23]. According to this model, the focus of attachment processes is largely on the developing child’s ‘organization’ of the self (i.e., self-regulation) via their use and processing of cognitive and affective information available within the caregiving environment [24]. It is important, however, to note that Crittenden [22] uses the term ‘cognitive’ to refer to the processing of very basic information regarding the temporal ordering of events, which does not necessarily require higher-order thought processes. A three-factor structure has been proposed, reflecting differing degrees of reliance and utilization of cognitive and affective information: 1) Type A (non-attending to affective information, reliance on cognitive information and expected consequences); 2) Type B (balanced processing and use of cognitive and affective information); and 3) Type C (reliance on affective information, non-attending to cognitive information and expected consequences) [21,24]. Crittenden [22] highlighted that both Type A (compulsive) and Type C (obsessive) strategies are associated with psychopathology owing to their tendency to overestimate the probability of danger and act in an unnecessarily self-protective manner. Similarities in these patterns of strategic information processing and behavior can be seen between Type A with avoidant attachment styles and Type C with anxious-ambivalent attachment styles.

A meta-analysis by Fraley [25] found attachment security to be moderately stable in the first 19 years of life. A subsequent large-scale study found that attachment security (measured categorically and dimensionally) was not stable from infancy to late adolescence; however, disorganized attachment at 15 months was predictive of preoccupied attachment at 18 years and security at both 24 and 36 months displayed an association with attachment security in late adolescence [26]. Given that a significant proportion of children and adolescents experiencing chronic and recurrent pains subsequently experience persistence of such into adulthood [27,28,29], the influence of enduring attachment patterns may be particularly important.

## 3. Attachment Framework and Vulnerability and Maintenance of Pediatric Chronic Pain

It seems likely that the complex early interactions between a child’s developing self-regulatory systems and their caregiver’s responses may impact on the vulnerability, onset and offset, or maintenance of chronic pain. The literature on each of these potential roles of attachment systems will be considered.

### 3.1. Attachment-Based Vulnerability Factors

A case could be made for arguing that attachment-based factors are more likely to render an individual vulnerable to the onset of a functional pain syndrome, with no readily identifiable pathology (e.g., migraines, headaches and abdominal pain), relative to other chronic pain conditions, such as those stemming from injury. Indeed, a large portion of the literature investigating attachment styles and pain has drawn conclusions based on patients with headaches or migraines (e.g., [15,30,31,32]) and functional abdominal pain (e.g., [33,34]). However, if acute physical threats are likely to activate attachment-based behaviors [16], it seems conceivable that attachment behavior patterns may contribute to an acute pain problem which has resulted from an injury or surgery transitioning into a chronic pain problem. To our knowledge there has not been any research investigating the role of attachment-based factors and the transition from acute to chronic pain. In the absence of any such guiding literature, the current paper will refer to chronic pain regardless of whether or not there is a known cause.

A number of attachment-based factors have been identified in the literature as potential vulnerability factors that may predispose an individual to developing chronic pain. These may include: (1) patterns of emotional expression (inhibitory or excitatory); (2) patterns of behavioral responding to threat; and (3) cognitive appraisal of threat. The literature pertaining to these factors will be reviewed. However, it is important to acknowledge that the studies have typically been cross-sectional, rendering it difficult to draw conclusions regarding cause and effect.

#### 3.1.1. Patterns of Emotional Expression (Inhibitory or Excitatory)

It has been proposed that attachment styles characterized by avoidance of emotional expression (e.g., insecure-avoidant, dismissing and fearful styles, or Type A attachment strategy) may predispose individuals to chronic pain conditions [35]. Children with this attachment style may have parents who respond to expressions of negative affect, including pain, by either withdrawing from their child or responding with displeasure or anger [35]. Children learn to inhibit verbal or nonverbal signs of distress, because they have found these to serve no useful protective function [23]. Attachment styles that are defined by excitatory self-protective mechanisms (i.e., insecure-ambivalent, preoccupied or Type C attachment strategies) are also likely to have implications for pain experiences. Children with this style may have parents who respond unpredictably. Consequently, the child may alternate between signaling various exaggerated expressions of negative affect (e.g., fear, anger, desire for comfort), with the aim of trying to get their unpredictable parent to respond [35]. In a sample of adolescents with somatization disorders, including functional pain syndromes, Kozlowska and Williams [35] observed that patients who utilized inhibitory or excitatory self-protective mechanisms comprised a disproportionately large percentage of the sample population compared to prevalence rates observed in healthy populations.

One possible mechanism to account for the relationships between inhibitory and excitatory emotional expression and pain responses relates to alterations in the body’s stress regulatory systems [35,36]. The cumulative adverse effects of chronic activation of the stress system are well documented (e.g., [37,38]), and are largely mediated by dysregulation of the hypothalamus pituitary adrenal (HPA) axis [39,40,41]. In adults, there is evidence that both anxious and avoidant attachment is associated with heightened reactivity of the HPA axis and autonomic nervous system reactivity to stress [41,42]. In light of the complex interactions between stress and multiple body systems (e.g., immune system, endocrine system), researchers have outlined a potential homeostatic role of increased pain sensitivity, and development of pain disorders characterized by central sensitization, in motivating restorative behavior for the body [43]. In this sense, pain is postulated to be one of a number of symptoms commonly associated with certain chronic pain disorders, e.g., fatigue, dyscognition [44], etc., that may function to promote survival of the organism through the promotion of quiescence. While it is difficult to delineate the direction of these relationships, research suggests the plausibility of both attachment deactivating and hyperactivating strategies contributing to dysregulation of the stress system within the body, and subsequently contributing to pain sensitivity.

The likely involvement of neurobiological processes in the manifestations of chronic or recurrent pains has been illustrated by studies of children with migraine. Insecure attachment has been found to be significantly more common in children with migraine compared to controls [31]. More specifically, the avoidant attachment style was found to have positive associations with migraine characteristics, including frequency, intensity and duration of attacks [31]. The authors highlighted the involvement of neurotransmitter patterns involved in the manifestation of attachment-related behavioral tendencies that also share links with the presence of migraine. Indeed, the literature supports the differential development of neural substrates associated with the caregiving environment and attachment styles [45].

#### 3.1.2. Patterns of Behavioral Responses to Threat

Patterns of behavioral responses to threat may have implications for how individuals respond to painful experiences and consequently the likelihood that acute pain experiences transition to a chronic pain condition. Certain attachment styles and methods of pain signaling may serve as effective and acceptable signals of potential danger or distress, increasing the likelihood of eliciting a protective caregiving response [46]. According to attachment theory, certain attachment styles and patterns of verbal and non-verbal pain communication may serve as effective signals of distress, increasing the likelihood of eliciting a protective caregiving response [46].

The caregiving environment that generates avoidant (fearful/dismissing or Type A) strategies typically results in limited signaling of negative affect in order to avoid characteristically aversive responses. However, it has been suggested that in certain circumstances, the attachment figure in this caregiving environment may tolerate pain (owing to it being understood as a physical symptom) as an acceptable signal of distress compared to fear, anger or sadness [46]. In these circumstances, signaling of pain may elicit a caregiving response serving to reinforce the behavior for any experienced distress [46]. Pain behaviors are also considered useful in ambivalent, preoccupied or Type C strategies, as pain signals that are sufficiently strong may successfully elicit desired caregiving responses from inconsistent caregivers [46]. This is partially supported by results from a study with an adult sample, where preoccupied, dismissing and fearful attachment styles were found to be associated with chronic widespread pain [47]. In this study, the authors suggested that the observed relationship may reflect an increased likelihood of insecurely attached individuals to communicate distress in terms of physical symptoms in lieu of emotional feelings [47,48,49].

#### 3.1.3. Cognitive Appraisal of Threat

The influence of attachment-related factors on cognitive appraisals of pain as a threat or a challenge affords another potential arena for contributing to vulnerability to chronic pain [50,51,52]. Pain appraisal is likely to have significant implications in the transition from acute to chronic pain states via its influence on the selection of coping strategies and subsequent adjustment to the pain experience, with known implications for a number of pain-related variables [33,53,54,55]. In Meredith et al.’s [53] Attachment-Diathesis Model of Chronic Pain, attachment-related primary appraisals of pain interact with secondary appraisals of the self (as equipped or not to cope; worthy or not of social support [56]) and of others (as available and adequate to provide effective support [57]) [52]. The attachment-diathesis model also emphasizes how such appraisals are influential in contributing to both the selection of coping strategies and subsequent emotional states, with threat appraisals likely to result in negative affect states [52,53]. The consequences of the utilization of particular coping strategies are likely to be mediated by contextual variables [52,58]; however, certain strategies have been found to be more commonly associated with poorer pain outcomes and negative emotional states, e.g., emotion-focused strategies that focus primarily on alleviating the experience of negative affect [52], such as pain catastrophizing or wishing [53]. Secure attachment has been shown to increase the likelihood of appraisal of pain as a challenge and subsequent utilization of problem-focused coping strategies such as seeking social support [50]. Conversely, insecure styles characterized by hyperactivating strategies have been shown to influence the appraisal of pain as more threatening [59,60], and in adolescents this was also related to anxiety, pain severity and depression [61]. In another sample of adolescents, Laird et al. [33] demonstrated that insecure attachment was influential in contributing to poorer health-related quality of life via its effect on cognitive appraisals and the subsequent employment of coping strategies.

In summary, there is sound theoretical basis, and some empirical evidence, for a range of attachment-based factors rendering individuals vulnerable to the development of chronic pain conditions, or vulnerable to the transition of acute pain conditions into chronic pain conditions. Patterns of emotional expression and behavioral responses to threat are of particular relevance and form within a developmental context of caregiver interactions. Moreover, the development of attachment style contributes to the manifestation of individual difference factors, such as cognitive appraisals of potential threats, with implications for responding to acute pain experiences. The significant limitation in this area of research is the reliance on cross-sectional rather than longitudinal designs, making issues of causality difficult to determine.

### 3.2. Attachment-Based Maintaining Factors

Not only do attachment-based factors have the potential to render an individual more vulnerable to the development of chronic pain, factors relevant to an individual’s attachment style may serve to maintain the chronic pain condition, regardless of the initial trigger or cause of the pain. Some of the factors identified as possible vulnerability factors for the development of chronic pain may equally serve to maintain a chronic pain condition. This symptom maintenance may occur in a number of ways. Insecurely attached individuals may be: (1) less able to manage the distress associated with pain; (2) more likely to use emotion-focused rather than problem-focused coping strategies; (3) less able to procure and maintain external supports; (4) less able to form therapeutic alliances; (5) less likely to adhere to treatment recommendations; or (6) more likely to evoke and perceive more negative responses from health professionals [53]. These factors may either serve to intensify the pain experience (e.g., through poorer ability to manage distress), or impede effective rehabilitation (e.g., through poorer therapeutic relationships) [62].

Some of these maintaining factors may be more pertinent to adult rather than pediatric pain patients, for example pediatric patients have less personal responsibility for procuring support from health professionals. Nevertheless, adolescents have an increasingly active role in accepting or rejecting potential sources of support. The process of actively seeking support inherently relies on an individual’s comfort with closeness to others, the belief that the self is worthy of support and that others are available to provide it [63], all of which are associated with secure attachment [53]. Individuals with dismissing and fearful attachment styles hold a view of others as unavailable or inadequately responsive, and are consequently less likely to acknowledge and seek help for distress related to pain [53,62]. Insecure individuals are therefore more likely to experience poorer pain outcomes.

An individual’s use of coping strategies, both in terms of cognitions as well as behaviors, has been found to be related to their attachment style and likely to impact on symptom maintenance. Individuals with secure attachment styles have been found to utilize more diverse, problem-focused and effective coping strategies [12]. In contrast, insecure attachment has been linked with more rigidity in selection of coping strategies and a tendency to employ emotion-focused or avoidant coping techniques [12,64]. Emotion-focused coping strategies have generally been found to be associated with poorer health outcomes in adults with chronic pain [65,66], potentially facilitated by low self-esteem and self-efficacy and the subsequent lessened desire to engage with rehabilitation and pain management programs [67].

In considering the notion of coping in children and adolescence, Schmidt et al. [68] have drawn a distinction between the appropriate selection and use of particular coping strategies, which has been referred to as ‘technical competence’, and the more interactive process of ‘adaptive coping’. Adaptive coping is conceptualized as more than a specific strategy, but as an interactive, developmental process, based on an awareness of context-dependent demands and effective employment of available resources. Attachment processes and interactions are likely to be particularly relevant to the ability to demonstrate competence in adaptive coping, which is considered the best predictor of long-term outcomes [68].

Adolescents with higher pain catastrophizing have been found to have more fearful and preoccupied attachment styles, which is consistent with the adult literature [50,55,69], and subsequently to have greater anxiety, pain severity and depression [61]. In addition to being associated with greater pain and poorer adjustment to chronic pain, pain catastrophizing may contribute to pain maintenance via affective inputs generating neurobiological pain-affect interactions [70]. This explanation is in accordance with the Attachment-Diathesis Model of Chronic Pain [53] which emphasizes factors in the maintenance of the role of attachment-related factors on producing negative emotion states that impact on poorer adjustment to and maintenance of chronic pain.

In summary, a range of attachment-based behaviors may contribute to the maintenance of chronic pain symptoms, most notably a range of interpersonal interaction styles, which when manifested in relationships with health care providers may contribute to the formation of barriers for the provision of effective treatment or adherence to treatment plans [62,71]. As outlined above, other attachment-based factors may also contribute to how a child or adolescent interacts with their primary caregiver and whether or not these interactions facilitate the adoption of clear communication and an active role in their own rehabilitation.

## 4. Attachment-Based Interventions and Chronic Pain

In light of the broad array of attachment-based factors potentially rendering individuals vulnerable to the transition from acute to chronic pain, as well as attachment factors potentially maintaining chronic pain, it is important to consider the best way that interventions can target these factors. Although a number of researchers have made theoretically-derived treatment recommendations for attachment-based interventions for chronic pain [53,72], such interventions have not been comprehensively outlined, let alone evaluated, within the pediatric chronic pain context. Hence, it is beneficial to consider attachment-based interventions that have been used and evaluated with children and adolescents targeting problems other than pain.

In 2015, a special issue of the journal *Attachment and Human Development* was devoted to articles describing attachment-based treatments for adolescents. These interventions have been used to increase security in the caregiver-adolescent attachment bond, while targeting a range of specific groups, such as depressed and suicidal adolescents [73], hard-to-reach youth [74], and pregnant adolescents [75]. An earlier review was carried out overviewing the attachment-based treatments for use with young children [76]. An underlying assumption for attachment-based treatments is that attachment and caregiving disturbances in some way contribute to the vulnerability, onset or maintenance of psychopathology or the problem in focus. The focus of attachment-based treatments with young children may involve modifying caregiver internal working models of self or others and increasing contingent and sensitive responding to their child’s signals and attachment needs. Attachment-based treatments with adolescents need to recognize that by adolescence individuals have become more active partners in maintaining the attachment bond [77,78,79]. Consequently, there are three possible treatment targets in attachment-based treatments with adolescents: (1) modifying the adolescent’s internal working model of self or others (especially their caregiver); (2) modifying the caregiver’s internal working model of self or others (especially their adolescent); and (3) promoting emotionally attuned communication between the caregiver and adolescent [80]. The caregiver’s ability to maintain a cooperative partnership with an adolescent is likely to be dependent on the caregiver’s ability to monitor their own emotions, clearly asserting their own positions, while validating and supporting the adolescent’s attachment and autonomy needs [79]. The caregiving environment is thus a necessary consideration in determining both the utility and appropriateness of an attachment-based intervention for pediatric chronic pain patients. As noted in the literature [72], if caregivers are unwilling or unable to engage in the therapeutic process, attempts at altering the working models of children or adolescents in isolation are unlikely to be effective and importantly may undermine the protective function of the established attachment strategy.

Although the attachment-based therapeutic targets outlined above are likely to be pertinent to the context of chronic pain, relatively little research has been done in this area. Numerous pain behaviors have been construed within an attachment framework, potentially rendering them as direct and indirect outcomes of attachment-based treatments. For example, individuals with insecure attachment styles have been found to describe their pain as more threatening and themselves as less able to deal with it [50,81]. Moreover, empirical studies with adults have found insecure attachment styles to be associated with lower trust and satisfaction with their physician [82], greater use of emotion-focused coping and less problem-focused coping [81], lower perceived social support [83], greater pain intensity and disability [14], greater pain-related distress [84], more physical symptoms [12], especially medically unexplained symptoms [13], and higher levels of pain-related stress, anxiety, depression and catastrophizing [50,55,85,86].

In the pediatric literature it has been demonstrated that among a preschool sample of four- and five-year-olds, representations of separation and pain experience were systematically related [87]. Moreover, children with relatively more ambivalent or disorganized attachments have been found to have a greater response to immunization injections and everyday pains [88]. Children with a more controlling attachment have also been found to take more time to calm down after an immunization injection, displaying greater anger [88].

It has been implied by attachment-based theorists that shifting the internal working models of the child/adolescent and the caregiver may help individuals develop more secure attachment styles. Notably, though, studies depicting associations between attachment styles and pain behaviors have generally been cross-sectional. Thus it is not known whether shifting the attachment style of a child/adolescent (and/or their caregiver) will result in changes in pain behaviors or pain outcomes. Longitudinal studies are needed to address such questions. Cognitive-behavioral interventions have, to date, been found to be the most successful treatments for pediatric chronic pain [89,90]. Some support has also been reported for acceptance and commitment interventions [91]. Theoretical and empirical work is needed to consider whether there is scope or value in utilizing attachment-based interventions for a certain group of pediatric chronic pain patients, or whether it is feasible to integrate attachment-based treatment components together with cognitive-behavioral therapy (CBT) or other evidence-based interventions. Despite there being numerous points of contact between attachment theory and the cognitive theory that underlies CBT (see [92] for a review), attachment theory and attachment-based interventions are largely unfamiliar to most cognitive-behavioral therapists. A potentially fruitful future research direction may be to screen pediatric chronic pain patients for insecure attachment styles and to determine whether these individuals benefit from interventions designed to shift the internal working models of the child/adolescent and/or the caregiver. Such interventions may be an adjunct to more established cognitive-behavioral therapies. McBride and Atkinson [92] have argued that attachment theory need not be used to generate new interventions, but may be used to better inform the practice and delivery of existing clinical modalities for the treatment of chronic pain. There is a precedent in incorporating aspects of attachment-based interventions with CBT for anxious adolescents [93]. Kozlowska and Khan [72] outlined a multidisciplinary, multimodal intervention for children and adolescents with medically unexplained pain, incorporating what is postulated regarding information-processing tendencies within the dynamic maturational model of attachment. The intervention highlighted the utility of considering observed deficits in the processing of affective information and addressing these in order to maximize the efficacy of any cognitive-based interventions [72]. The subject intervention reportedly facilitated reductions in pain intensity and improvements in school attendance [94]. Further research is needed in this area within the pediatric chronic pain context.

The inclusion of parent-based treatment components in pediatric chronic pain programs has widely been accepted as being of considerable importance [95]. The efficacy and value of such parent-based interventions may in part be understood from an attachment framework. It is well established that children whose parents engage in more protective behaviors (e.g., excusing their child from the usual roles and responsibilities) have greater functional disability [96,97], more school absences and impairment [98], and greater healthcare utilization [99]. Parental protective behaviors have been found to occur when parents lack confidence in their child’s ability to cope with their pain [100]. The context of a medical condition is likely to activate a unique set of caregiver attachment behaviors, motivated by a desire to maintain ’safety [16]. However, when a condition is chronic, it is not helpful for caregivers to remain in a protector role. Adolescence is a time when the need for security must be balanced with an increasing need for autonomy, and a highly protective caregiver is likely to minimize an adolescent’s opportunity for autonomy and practice with problem-solving. The goal of emotionally-attuned communication between caregiver and adolescent is likely to be key to providing adolescents with a secure base from which to take ownership of their pain problem and to actively engage in its management, while knowing that they have caring support when needed.

## 5. Challenges and Future Directions for Research

Although a small number of papers have outlined the potential theoretical relevance of the attachment theory to the chronic pain context [46,53,62], more empirical work is needed to investigate theoretically-derived hypotheses. Questions related to causal direction remain elusive, largely due to the difficulty in carrying out longitudinal research with children who will at some point in the future develop chronic pain. However, even if such longitudinal research were carried out, the causal direction between attachment style and chronic pain might not be unidirectional. Although insecure attachment styles may render individuals vulnerable to developing chronic pain, the pain experience is thought to be likely to bring out an individual’s characteristic attachment behaviors, suggesting that a cyclical relationship may exist.

Clinical research investigating attachment styles and treatment outcomes among pediatric chronic pain patients may prove fruitful. There is reason to predict that individuals with insecure attachment styles have poorer treatment outcomes with established evidence-based interventions, perhaps due to lower trust and engagement with health professionals [82] or less use of problem-solving strategies [81]. The next step would be to investigate whether particular patient groups, perhaps differing in attachment variables, would benefit from evidence-based interventions utilizing a more specific attachment focus, or from incorporating an additional attachment-based adjunct treatment component. This would require screening pain patients for attachment-based variables; preliminary investigations are needed to determine which domains of attachment are most closely related to chronic pain outcomes. Self-report questionnaires, such as the Inventory of Parent and Peer Attachment [101], tap into more conscious, strategic aspects of attachment, but may overlook more automatic aspects. Behavioral and observational measures may tap into automatic as well as strategic aspects of attachment, but are more resource-intensive to administer and to code.

Another clinical question which warrants investigation is whether individuals with particular attachment styles are more likely to transition from an acute pain condition to a chronic pain condition. If one is to accept the claim that an individual’s attachment style is likely to be activated by a physical threat such as injury or surgery, there would be grounds for hypothesizing that an insecure attachment style may confer a risk for acute or post-operative pains developing into a chronic pain condition. There have been relatively few investigations into potential risk factors associated with a transition from acute to chronic pain in children [102,103], and attachment style has not been considered in these studies.

Finally, it is worth acknowledging that the complex relationship between attachment and pain has also been considered from theoretical perspectives other than attachment theory. Evolutionary perspectives have been used to account for underlying neural similarities between social isolation/exclusion and pain [104,105]. According to this perspective, social isolation, just like physical pain, is designed to trigger an internal alarm, warning of a situation that may be harmful to survival. Just as the unpleasantness of pain prompts an individual to engage in behaviors intended to minimize physical harm, evolutionary pressures may have resulted in social pain or intense negative feelings serving as an indicator of an impending, and potentially dangerous separation or exclusion [106]. Neuroimaging techniques have afforded these theories some support (for a review see [104]).

## 6. Conclusions

Although there has been relatively little literature relating the attachment framework to the pediatric chronic pain context, there is merit in considering this relationship from a theoretical, empirical and clinical perspective. A range of chronic pain behaviors have been theoretically accounted for by an attachment framework (e.g., [46,62,107,108]); however, the empirical literature to date has been sparse. A number of attachment-based factors have been identified as possible vulnerability factors, predisposing individuals to the onset of chronic pain. These include preoccupied, dismissing and fearful attachment styles, as well as Type A and Type C attachment strategies as defined by the Dynamic Maturational Model. Attachment factors may also serve to maintain chronic pain conditions through a variety of mechanisms, such as poor distress management, maladaptive coping strategies, poor utilization and compliance with healthcare services, and limited access and utilization of social supports. More empirical work is needed before attachment-based treatment targets can be applied to the pediatric chronic pain context. For example, consideration should be given to whether screening chronic pediatric pain patients for particular attachment styles may help identify patient subgroups that might benefit from interventions with a particular attachment focus or as an adjunct attachment-based component to the primary therapeutic approach.
